# Reflections and Projections: The Next 50 Years of Priorities in Nursing Education

**DOI:** 10.1111/jan.70591

**Published:** 2026-03-25

**Authors:** Fiona Timmins

**Affiliations:** ^1^ School of Nursing, Midwifery & Health Systems University College Dublin Dublin Ireland

Over the past 100 years, the nursing profession has undergone a profound transformation in terms of the approach to the educational preparation of nurses. Once educated and trained at the individual hospital level, largely to suit health service needs, preparation for entry to the nursing profession now mostly rests within the 3rd level sector and universities. While this transition of the preparatory education of nurses into the university sector is not uniform globally, it has laid the groundwork for the ongoing professionalisation of nursing, something which initially gained momentum in the early part of the last century.

This commentary reflects on some key milestones in this profession's evolution from an education perspective and outlines some critical areas requiring ongoing attention to meet the needs and demands of the 21st century and beyond. As we commemorate the 50th anniversary of the *Journal of Advanced Nursing*, this commentary offers a retrospective view of some key developments in nursing education and a forward‐looking analysis of a sample of critical issues shaping the future. Recommendations are offered to support appropriate progress in nursing education.

This brief outline of the historical development of the nursing profession reveals some points that inform and influence current debates in the education sector. Given the diverse and inconsistent manner of the educational standardisation, regulation and education of nurses globally, and to explore important key concepts in a little more depth, some key elements of this professionalising journey will now be discussed with a particular focus on Ireland. This will be helpful to inform suggestions for the future direction of travel that will have application more globally.

## 1 The Professionalisation of Nursing

The professionalisation of nursing is a relatively new development globally. At the same time, in most countries, nursing has a long history. Communities have historically cared for their sick. However, there was often no standardised approach to nursing, and nurses were unregulated. At the same time, there is often limited information about the nature and evolution of the role of nurses historically, as this was not always accurately recorded or documented. For example, in Ireland, nursing has ‘a long and distinguished history’; however, much of this legacy is ‘invisible’ (Treacy 2005, 42).

Nonetheless, what is known is that due to advances in medicine and science, the latter part of the 19th century heralded more formalised and uniform approaches to nursing and nurse education across many parts of the world. Resultantly, major initiatives occurred over the next century that advanced the professionalisation of nursing (Fealy 2006a). The hallmarks of professionalisation include holding a monopoly on certain jobs, degree or certification and regulatory mechanisms. This journey occurred in nursing through an initial standardisation of the education of nurses that occurred within mainly urban hospitals. This led to clear mechanisms to determine the correct preparation for a person to be permitted competent to hold the title of nurse. The development of national registers for nurses and organisations that oversaw these standards further cemented this and determined who could have their name entered as a nurse on the register. This standardisation and regulation also led to nurse certification. In the past 50 years, pathways opened to enable nurse certification to be advanced to diploma and degree level. Increasingly now, nurses are advancing their education and training at postgraduate and PhD level. In some countries, such as Ireland, this professionalisation also paved the way for the advent of advanced and specialist practice roles for nurses.

It is worth noting that both nursing regulation and certification, including the advancement towards degree‐based education, post graduate education and advanced and specialist practice occurred and are occurring at different rates throughout the world. While most countries internationally hold a register of nurses, the extent to which these regulatory bodies have powers to regulate ongoing competence requirements and nurses' fitness to practice varies. Moreover, while certification is usual, the academic requirement to enter the nursing register varies. In some countries this is at degree level, and in others at diploma or certificate level. In some cases, there exist various tiers of nurses who can register and practice. The United States of America (US), for example, one of the first areas to provide degree‐based education for nurses, has a two‐tiered nurse admission system. Within this system, there are two main pathways to becoming a registered nurse. Firstly, a two‐year Associate Nursing Degree (AND) from a community college and secondly a four‐year Bachelor in Nursing Science (BSN) from a university. Graduates from both are eligible to take the NCLEX‐RN (National Council Licensure Examination for Registered Nurses) (NCSBN) to become Registered Nurses (RNs). Regarding advanced and specialist nursing practice, this is well developed in many European, US and Canadian, and some Australasian regions, but varies in approaches, regulation and certification.

### 1.1 Advancing Nursing Knowledge and Practice

The formalisation of the education of nurses across Europe and other parts of the world, from the 19th century onwards, was due in part to major advancements in developments in medicine and technology, the rise of hospital‐based medicine, and religious order nursing (Fealy 2006a). Indeed, the religious orders founded many hospitals in Europe and the US and provided the required education for their nurses therein. We can observe the influence of religious orders in Ireland, for example, where, in the 19th century, Irish women founded and led religious congregations that cared for the sick (Treacy 2005). Most prominent among these were Catherine McAuley, who founded the Sisters of Mercy in 1831, and Mary Aikenhead, who founded the Sisters of Charity in 1815 (Fealy 2006a). The Sisters of Mercy later contributed to nursing the sick in the Crimean War (1854–56) alongside Florence Nightingale (Treacy 2005). Because of this history and the nature of the work, nursing became associated with a vocation or ‘calling’. However, the development of modern secular‐professional nursing in Ireland, after Nightingale, occurred as an Anglican social reform movement led by educated middle‐class lady superintendents of nursing, who had trained in the elite charity hospitals in London and elsewhere in the kingdom (Fealy 2006a). Occurring after 1870, the reform involved the introduction of hospital probationership training schemes and associated reforms in sanitary arrangements and hospital management.

During this period, many hospitals in Ireland (when it was still part of the United Kingdom) provided nurse training schools. However, as there was no official register or regulatory body for nurses, individuals could work as nurses without having undergone formal training (Fealy 2006a). Thus, a vociferous and sustained campaign emerged among UK and Irish nurses at the time towards achieving professional regulation (Fealy 2006b:94). The goals of professional regulation were state registration for nurses and standardised education (Fealy 2006a). In Ireland, a notable campaigner for this was Margaret Huxley, Matron of Sir Patrick Dun's Hospital (Fealy 2006a, 2025).

State registration for nurses occurred in the UK in 1919 with the enactment of the Nurses Registration Act (Fealy 2006a). This was a source of great pride for nurses that was celebrated at the time. Although Ireland was part of the UK at the time, the Act did not apply to Ireland, and it was only after urgent representations from nursing leaders in Ireland that the Government at Westminster passed the Nurses Registration (Ireland) Act in the same year. The Act provided for the establishment of the General Nursing Council for Ireland, the first regulatory authority for nursing in Ireland. The Council regulated nursing from 1920 to 1950, a period that coincided with the first thirty years of the newly independent Irish Free State (Fealy 2025). As part of the planning to develop a national public health service along similar lines to those in Britain, the Irish Government reconstituted the Council as An Bord Altranais (the Nursing Board) in 1950, and in the process, incorporated the Central Midwives Board into the new Nursing Board. Nursing regulation was further enhanced with the passing of the Nurses Act 1985, which provided for the introduction of a ‘Live Register’ in 1987 and a ‘Fitness to Practice Committee’ in 1986.

Hospital‐based nurse education continued in Ireland, and major changes to curricula occurred in the 1980s and 1990s due to changing European directives (Fealy 2006b). This meant that each hospital was required to provide a strict number of prescribed hours of theoretical instruction and specific clinical instruction in key specialist areas, such as maternity care and mental health (Fealy 2006b). Later, in 1994, the Irish Nursing Board recommended the separation of nursing education and nursing service to be achieved through the introduction of a common core model of training to be developed under joint validation arrangements with universities, with a Diploma in Nursing awarded on the completion of a three‐year training programme (Fealy 2006b).

However, the most fundamental changes took place following the implementation of the recommendations of the Commission on Nursing, established in 1997 and published in 1998. Its recommendations were far reaching and included changes to the management and development of the profession, in addition to the key recommendation for the award of a four‐year honours bachelor's degree at the point of entry to professional practice. The first cohort of undergraduate students commenced training in 2002 after which nursing in Ireland was established as an all‐graduate profession. The Commission on Nursing 1998 heralded a professionalising agenda in Irish nursing and midwifery. As an all‐graduate entry profession, the central pillars of career progression were increased clinical career pathways and increased emphasis on and support for Irish nursing research.

This change of perception and indeed status of the nursing student from *worker* to student was an important outcome of these developments. Research in both the UK and Ireland had already determined that the historical hospital‐based certificate programmes that student learning was hampered by being an employee. Students did not feel autonomous from the hospitals. They were afraid to speak up and tended to keep their heads down and get on with the work. In Ireland for example interviews with nursing students revealed that as workers they ‘did not challenge the status quo, rather they got on with the work and hoped for a good report [on their performance]’ (Stakelum 2006:14).

This was an important discourse that resonated across many research study findings that informed many nurse educators' views that a university education for nurses was a necessity. It was recognised that while hospital‐based education served its purpose in the 1800s and 1900s, the landscape of health care provision had changed radically in the intervening century. Ultimately, a technologically and medically advanced health system, in the context of an aging population, global trends of health promotion and holistic person‐centred care together with the evidence‐based practice (EBP) movement meant that there was both a welcome and indeed a necessity for the opportunities that a university‐based education could afford. In particular, there were greater opportunities to learn about leadership, research, health promotion, psychology, sociology, and anatomy and physiology from experts in a way that was not available in the small hospital‐based schools. Advancing nurses' knowledge about research and requiring their teachers to be research active (a university requirement) were particularly important because the EBP movement required all nurses to be able to critique and use healthcare research, and also because research activity in a discipline serves as a proxy measure of its academic success (Treacy 2005).

At the same time, the higher education sector in Ireland acknowledged and retained the close connections with the health services. The nursing programmes are delivered in partnership, with students spending at least 50% of their educational time undertaking clinical placements (Ryder et al. 2025). The importance of this connection and collaboration was provided for within the recommendations of the Commission of Nursing (Government of Ireland 1999). The Irish regulatory body monitors this engagement through the provision, regulation and oversight of standards for nurse education that need to be complied with by the relevant 3rd level establishments.

2026 marks the 20th anniversary of the first nursing graduates in Ireland. At the same time, developments in advanced and specialist nursing (SN) practice mean there are now more than 700 Advanced Nurse Practitioners (ANP) working across a range of health services, making up more than 10% of the total workforce (Nursing and Midwifery Board of Ireland 2024). Evaluation of these services revealed a very positive impact on health service delivery and patient experience (Brady et al. 2020). The ANP is also experienced as a highly autonomous and satisfying role (Duignan et al. 2024). The educational support for SN and ANP is provided by the 3rd level sector, in close collaboration with the health services. Higher diploma and master's degree (respectively) are standardised requirements for these roles that are regulated by the NMBI. Consequently, the 3rd level sector has a vibrant postgraduate suite that supports and educates nurses in many areas of practice and specialism, facilitating SN and ANP roles, but also advancing knowledge and skills across a range of areas including leadership and management. Irish nurses have also become advanced in research and scholarship, having propelled themselves from having a very minor representation in peer‐reviewed publications (less than 800 in 2002) to 10,965 in 2025. As publication output is a key element of the measurement criteria for university rankings, it is not surprising therefore that three Irish nursing schools are within the top 50 rankings worldwide. Curriculum reform and modernisation in Ireland is ongoing, with a new curriculum planned for 2027 with increased focus on community health, health promotion, and digital competencies.

### 1.2 Challenges for the Advancement of Nursing

Continuous advances and developments in technology and medicine, together with a continuously aging population presenting with increasingly complex co‐morbidities means that the advanced education and skills of nurses, particularly the SN and ANP, provide a particular strength to health service delivery in Ireland. However, one of the greatest challenges facing the profession is difficulties with retaining the full complement of nurses needed to provide frontline services. These workforce issues are not unique to Ireland and are indeed a concern globally (World Health Organization 2020). While nurse researchers and health services in Ireland are actively supporting advancements in workforce planning to address this (Brady et al. 2025), in consultation with international colleagues, attention is also being drawn to the need for the potential to increase the nursing student population to address these gaps.

This situation is challenging as numerical increases place an incumbent burden on the resources of university and health services who are not consistently supported to address the increased demands. Supporting the education of nursing students in the healthcare environment involves more than simply adding them as workers; it requires significant resources. As they are not counted as staff within the staffing ratios (termed ‘supernumerary’ status) for most of their programme, they require individualised support, education, assessment and attendance monitoring. This is something that is being consistently overlooked in the rhetoric about increasing student places.

Workforce concerns are widespread, and this has also led to a popular suggestion for nurse education to return to a form of the hospital‐based education (termed apprenticeship nursing), where they would spend more time in clinical practice (i.e., university holidays reduced), receive a low pay, and effectively be workers from the outset. This notion that additional nursing students can be simply ‘added’ to the system as workers seems to hark back to the historical trend of nurses learning on the job. Unfortunately, this proposal often receives a weight of public support, largely because those outside of the profession may retain notional ideas of nursing as a vocation, and because of the legacy of negative discourse around academic nursing education has accompanied this professionalisation. Moreover as nursing has been historically perceived as women's work and often treated as a vocation rather than a professional career this perception can serve to influence how nursing is viewed in terms of its societal value and professional respect, which in turn can lead to the profession being more susceptible to political changes that might diminish its status, pay, or educational requirements. Indeed the International Council of Nurses (ICN) commenting on a World Health Organization (2022) report that highlighted gender differentials in pay within healthcare commented that ‘the ICN has been calling out the historical, chronic and deep‐rooted undervaluing and underfunding of feminised work, with nursing being a prime example’ (International Council of Nurses 2022, 1).

### 1.3 Negative Discourse Around Nursing Education‐ The Anti‐Academic Debate

In Ireland the move to graduate status was met with some ‘suspicion’ and scepticism in Ireland (Fealy 2006b:103) although it was mostly favourably received by the nursing profession. This negative discourse relies in part upon a public rhetoric, played out for example in the UK public media, that is politically motivated, that apportions blame on the nursing profession, and specifically the education of nurses, for highly published healthcare scandals involving poor standards of care. This media scepticism has been called out as targeting the nursing profession as ‘scapegoat’ for ‘deficiencies’ in the health care system (Fealy and Newby 2005). Indeed, the phrase ‘too posh to wash’ was coined in the UK (Beer 2013) for example, in response to the Mid Staffordshire enquiry, which found evidence of poor care (Francis 2013). Serious lapses in care quality have been revealed in health care scandals across the globe. And while substandard nursing care were not the sole contributing factor, within the context of such scandals, nurses are viewed in these contexts as having let down the nursing profession. Indeed, the negative discourse that arose about ‘academic nursing’ (Beer 2013) heralded an inquiry into the contribution of nurse education in the UK (Willis 2012), which overall found that no fault lay with university‐based education and that it should continue.

## 2 The Next 50 Years of Priorities in Nurse Education

From the move to university‐based education and the emergence of CNS and ANP roles to the growing impact of nurse‐led research and policy, the profession has positioned itself as an important influencer of the health system globally. However, ongoing challenges, including workforce shortages, advancing technology, increasingly older populations and a reduced faculty pipeline, mean that the profession needs to consider priorities that would enable it to be well positioned to continue to support the health system in an optimal manner. Of most imminent and important of these is supporting the global nursing workforce.

### 2.1 Supporting Global Nursing Workforce Enhancement

The shortage of nurses is set to increase with a projected deficit of over 5 million nurses by 2030 (World Health Organization 2020). Addressing workforce issues through increasing the attractiveness of the nursing profession may require more creative use of resources. The advent of university‐based nurse education has meant a stepping down of previous incentives that were a feature of hospital‐based education, namely free education, subsidized food, accommodation and a salary. In many parts of the world nurse education now involves university fees with no stipend. These original financial incentives rendered nursing an attractive career proposition. The opposite is also true. Nursing students' contribution to nursing work without pay can be off‐putting for some.

From this perspective, the suggestion to reverse disciplinary progression and move back nursing to an apprenticeship model is understandably attractive. It appears to add the missing financial incentive. However, this type of professional step back is unprecedented across the university sector. The positioning of nursing students in a work environment, exposed to the variety of out of hours working across a 12‐month period (with no university holidays) and expecting them to learn with limited educational support structures, is exceptionally risky. This approach worked in the 1800s and early 1900s in an era that predates most modern approaches to medicine and healthcare, including basics such as understandings of infection disease transmission and the discovery and use of antibiotics.

Nonetheless, the argument gains weight from naïve views of nursing as a vocation, inappropriate apportioning blame to the nursing profession to deflect accountability in national healthcare scandal (Fealy and Newby 2005), workforce shortages, and dissatisfaction among nursing students with pay and conditions. It is also positioned as a cost‐cutting exercise. Universities in the UK that supported nursing apprenticeships pilots were provided with fees for students, from the health sector, that significantly undercut the cost of educating students. Indeed, a report by KPMG (2018), commissioned by the Council of Deans in the UK, confirmed the substantial costs related to running clinical‐based programmes, including nursing. To undercut these costs is an incredible risk to the integrity of the programme and the support that students need to succeed. There are also many logistical issues with the financial model. Indeed, there are growing concerns about the long‐term feasibility and sustainability of this approach (Committees Parliament UK 2018). That said, students appear to positively review the programmes; the financial rewards are beneficial (Cushen‐Brewster et al. 2022). They do, however, find they are overburdened and struggle with balancing their role as student and worker (Cushen‐Brewster et al. 2022). Overall, it appears that there are high costs to the health services, with the university sector undercut, so overall no realistic cost saving (Committees Parliament UK 2018). There is also a very high attrition rate (at least 25%) (Cushen‐Brewster et al. 2022).

Another alternative approach that has received less consideration is that the health sector simply sponsors the current university degree students. In countries such as Singapore, for example, the health services provide substantive scholarships that support the student's living expenses and university fees. As part of this arrangement, students agree to work for the health services for defined periods. There are also options to self‐fund to avoid this pay back. The integrity of the degree programme remains intact.

Preserving the integrity of the professionalisation of nursing and advancing the profession requires a continued commitment to high quality university‐based education that permits students to experience university life and become educated alongside and in a similar fashion to other health professionals. Attempts need to be strengthened to increase the value placed on the university contribution in preparing competent graduates, but also supporting the education of nurses to advance their practice.

More also needs to be done to ensure adequate numbers of future nurse educators. Bond and Jackson (2025) recently highlighted the shortage of future nursing academics, emphasising its potentially detrimental impact on nursing education and the broader healthcare system. They argue that this is a systemic challenge requiring comprehensive, long‐term solutions. Bond and Jackson advocate for strategic investments in faculty development, enhanced support structures, and policy reforms that prioritise the sustainability of the nursing academic workforce. The costs of preparing for a university nursing academic role can be substantive. PhD fees can be costly, and due to minimal salary differentials, there is little financial incentive to take on these costs and move from nursing roles in healthcare. This is a particular challenge in Ireland, where strategic incentives such as the PhD scholarship Schemes are serving to address some of this imbalance.

## 3 Conclusion

The twentieth century reflected an accelerated advancement of nurses, with a clear professionalising agenda, uniquely driven from within the profession. The motivation, dedication and commitment of nurses and nurse educators are clear. The role of the nurse has extended and advanced, with nurses providing high level evidence‐informed care. Internationally, many nurses are working as ANPs and CNSs providing individualised, autonomous and evidence‐based care across various health care settings that they find rewarding and patients appreciate. The prescriptive authority of ANPs and their role in primary care delivery has been vital in improving access to healthcare services. These roles have also contributed to cost‐effectiveness and patient satisfaction, particularly in relation to primary care provision and chronic disease management.

The future requires continued support of these roles within the 3rd level and university sector, along with increased standardisation and synchronisation of these roles globally. Increased understanding of the unique and complex contribution of nurses to the health services is needed at a public level, to offset rhetoric and perceptions of nurses that are reflective of historical foundations rather than the nurse that has evolved due to modern medical and technological advancements. This strengthening of nursing's identity needs to develop both nationally and globally to ensure that nursing consistently has a political voice and public representation that ensures the continued growth and professionalisation of nursing.

To support continued advancement and to respond to current health care challenges and trends, the provision of nurse education needs to continue within the 3rd level and university sector. This will enable nurses to continue to engage in a broad and robust curriculum that enhances their knowledge and skills, particularly in relation to leadership and management, digital and AI competencies, community health, environmental and cultural issues. These nurses need to be supported in their clinical learning environment in the health care services, through appropriate educational support. Plans that enable nursing students to learn as apprentices need to be thoroughly and realistically evaluated from a financial perspective, but also from the potential negative impact on the professionalisation and perception of nursing before such approaches become more widely adopted globally. Moreover, financially incentivising students taking university‐based degree and diploma programmes needs serious consideration.

It is important to celebrate past nursing achievements, but it is also important to confront pressing challenges. The professionalising of nursing has benefited global health in an important and meaningful way. The last 100 years have demonstrated the extraordinary capacity of nurses to adapt, lead, and innovate. From bedside to boardroom, the profession has matured into a critical pillar of the global health workforce. Yet the work is far from done. Our task as educators, practitioners, and leaders is to prepare nurses who are not only clinically proficient but also socially accountable, technologically literate and globally conscious. The next 50 years will define whether we are able to build a truly equitable, resilient and nurse‐led healthcare future. Let us rise to that challenge with humility, hope and unwavering resolve.

## Funding

The author has nothing to report.

## Conflicts of Interest

The author declares no conflicts of interest.

## Data Availability

Data sharing not applicable to this article as no datasets were generated or analysed during the current study.
